# WISH-R– a fast and efficient tool for construction of epistatic networks for complex traits and diseases

**DOI:** 10.1186/s12859-018-2291-2

**Published:** 2018-07-31

**Authors:** Victor A. O. Carmelo, Lisette J. A. Kogelman, Majbritt Busk Madsen, Haja N. Kadarmideen

**Affiliations:** 1 Quantitative and Systems Genomics Group, Department of Bio and Health Informatics, Technical University of Denmark, Kemitorvet, Building 208, 2800 Kgs. Lyngby, Denmark; 2 Animal Breeding, Quantitative Genetics and Systems Biology group, Department of Large Animal Sciences, Faculty of Health and Medical Sciences, University of Copenhagen, Frederiksberg, Denmark; 3 Danish Headache Center, Department of Neurology, Rigshospitalet Glostrup, Nordre Ringvej 69, 2600 Glostrup, Denmark; 4 Institute of Biological Psychiatry, Mental Health Centre, Sct. Hans, Roskilde, Capital Region of Denmark Denmark

**Keywords:** Epistasis, Networks, GWAS, Complex traits, WGCNA

## Abstract

**Background:**

Genetic epistasis is an often-overlooked area in the study of the genomics of complex traits. Genome-wide association studies are a useful tool for revealing potential causal genetic variants, but in this context, epistasis is generally ignored. Data complexity and interpretation issues make it difficult to process and interpret epistasis. As the number of interaction grows exponentially with the number of variants, computational limitation is a bottleneck. Gene Network based strategies have been successful in integrating biological data and identifying relevant hub genes and pathways related to complex traits. In this study, epistatic interactions and network-based analysis are combined in the Weighted Interaction SNP hub (WISH) method and implemented in an efficient and easy to use R package.

**Results:**

The WISH R package (WISH-R) was developed to calculate epistatic interactions on a genome-wide level based on genomic data. It is easy to use and install, and works on regular genomic data. The package filters data based on linkage disequilibrium and calculates epistatic interaction coefficients between SNP pairs based on a parallelized efficient linear model and generalized linear model implementations. Normalized epistatic coefficients are analyzed in a network framework, alleviating multiple testing issues and integrating biological signal to identify modules and pathways related to complex traits. Functions for visualizing results and testing runtimes are also provided.

**Conclusion:**

The WISH-R package is an efficient implementation for analyzing genome-wide epistasis for complex diseases and traits. It includes methods and strategies for analyzing epistasis from initial data filtering until final data interpretation. WISH offers a new way to analyze genomic data by combining epistasis and network based analysis in one method and provides options for visualizations. This alleviates many of the existing hurdles in the analysis of genomic interactions.

**Electronic supplementary material:**

The online version of this article (10.1186/s12859-018-2291-2) contains supplementary material, which is available to authorized users.

## Background

High throughput genotyping data have been used extensively in many contexts to explain phenotypic variation of complex traits in a wide range of Genome Wide Association Studies (GWAS). GWAS can however, only partially explain observed phenotypic variation [1], and phenotypic variation has been shown to eclipse genotypic variation in the same population [2]. For example, in a large study of inflammatory bowel disease (IBD) only 8.2–13.1% of the variance in disease liability was explained using GWAS [3]. Several factors can explain the missing heritability of complex traits [4], but one often overlooked aspect is epistasis which can contribute to genetic variation in complex traits. Epistasis can have at least two definitions [5], but here we mean the use of genome-wide multi locus genetic interactions to predict phenotypic variation. Epistasis commonly affects phenotypes [6] and is observed in type 1 and type 2 diabetes [7, 8] and IBD [9] risk loci. Thus, quantification of epistasis can improve our understanding of causal genomic variation.

Calculation of epistasis is a computational challenge, even on modern computing facilities. To calculate first order epistatic interactions, that is, interaction between pairs of genotypes, of N loci, it is necessary to do minimum $\sim \frac{N^2}{2}$ estimates. In the case of a 700 k SNP array, this leads to and order of 2.5 × 10^11^ computations and a large memory consumption, both generally intractable. Therefore, it is important to have strategies to properly filter and reduce input data dimensionality. In general, when analyzing a specific trait it is assumed that most variants are not causal or associated with the trait. Furthermore, many variants will be in high linkage disequilibrium (LD) when using modern high-density genotyping arrays, meaning that their resulting interactions will be highly correlated. Thus, it is not only necessary to filter the input space due to computational issues, but also meaningful from an analysis perspective.

Beyond computational issues, interpretation of epistatic interactions can also be difficult. As the number of tests increases to the square of the input, multiple testing correction will be very stringent, making it difficult to rely on individual interactions. From a biological perspective, it would be useful to look at groups of genes and pathways instead of focusing on single variants. One way of integrating and combining signal from multiple sources is to use network-based strategies. Using networks-based methods is a useful and successful approach in identifying pathways and genes related to complex traits [10, 11]. A widely used method for this is the WGCNA method and R package [12]. WGCNA is designed for gene expression data, creating networks of co-expressed genes. To take advantage of this feature in a genomic context, the WISH (Weighted Interaction SNP Hub) method was developed by Kogelman and Kadarmideen [13]. WGCNA is built on the assumption that genes that are co-expressed are functional in similar pathways. WISH extends this hypothesis into the assumption that loci that show epistatis are functionally related. WISH calculates epistasis and creates biological networks based on said interactions. The goal is to identify modules of interacting loci that affect a phenotype or complex trait of interest.

We have developed an efficient and easy to use R package based on the WISH method and added several features including LD based data dimensionality reduction. Using input genotypes and a phenotype the WISH R package filters the data, calculates genome-wide epistatic interactions and generates biologically meaningful networks.

## Implementation

### Inputs and filtering

**Fig. 1:**
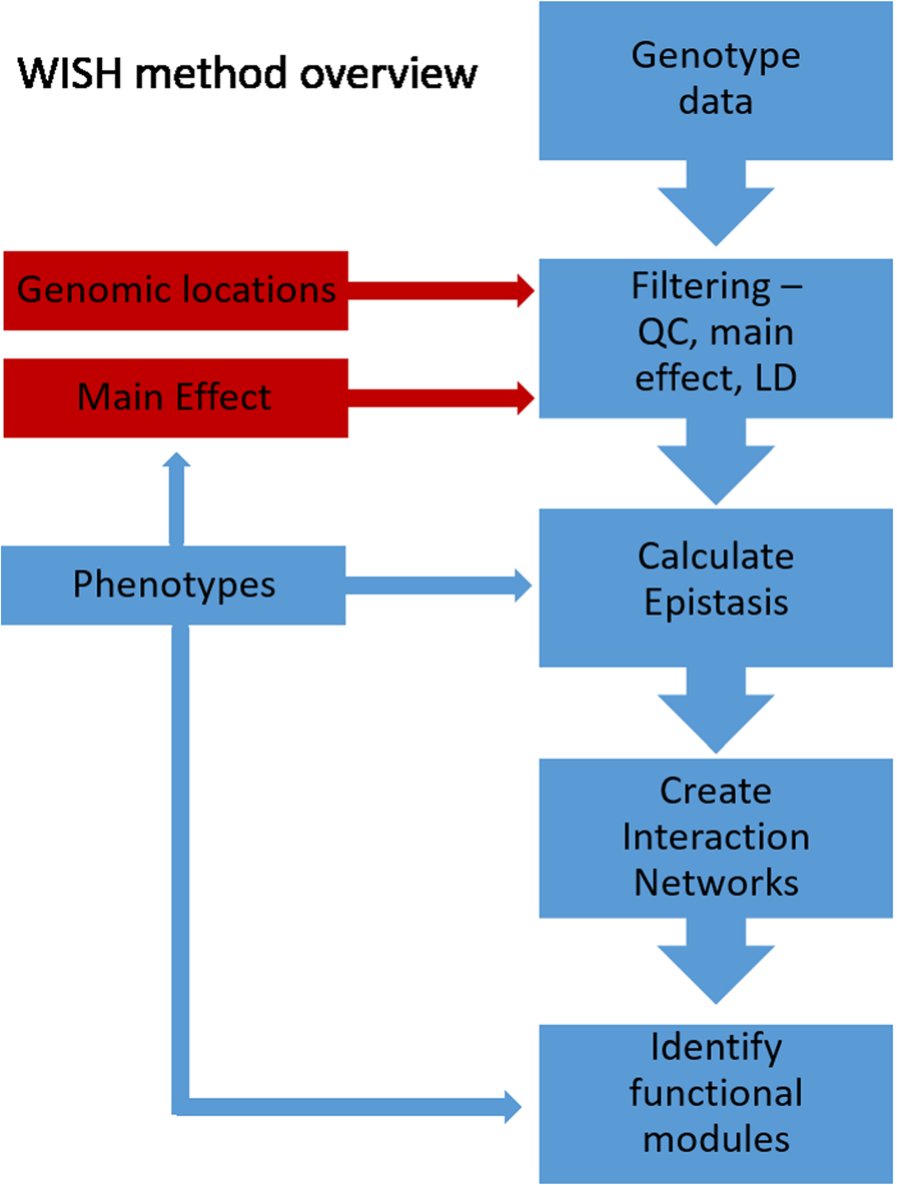
Overview of the package pipeline and workflow. The method only requires phenotypes and genotype data to run. The boxes in red are optional but recommended. The genotype data should be input using the PLINK ped and tped format [14]. Phenotypes can be either continuous or binary. The WISH method can be separated into three overall parts: QC and data filtering, calculation of epistasis and network and module generation. The QC should be similar to a standard GWAS based on call rates and minor allele frequency. An additional step can be done to filter based on LD, which is built into the package. The calculation of epistasis is the most computationally heavy part and is fully parallelized. The network and module construction part is based on converting the epistatic coefficients into correlations and running the WGCNA pipeline, which is integrated into the WISH package

The WISH R package is based on the WISH method [13]. The input files required for the method are a pedigree (ped) and a transposed ped (tped) file, both following standard PLINK format [14]. The overall workflow is shown in Fig. 1. We highly recommend that the raw phenotype data are adjusted for fixed effects and covariates such as sex, age etc., before running genome-wide epistatic model, as they need to be estimated only once. This is done in simple linear regression model fitting all non-genetic fixed effects and, obtaining estimated effects and correct the phenotypes accordingly. We recommend running a simple GWAS on your data first, and then filtering input SNPs based on significance. This helps reduce data dimensionality, as variants with no main effect are unlikely to have epistatic effects, as these would show up at least partially in the main effect estimation. However, we do not recommend strict filtering, as the efficiency of our implementation allows testing of a large number of interactions, as discussed further below. This means we recommend including as many variants as feasible depending on the available computational power.

Once a suitable set of variants has been selected it is possible to further filter the data by using LD. Variants in high LD are redundant and will lead to the same nearly identical models being estimated several times. In the context of WISH-R we are not interested in a probabilistic measure of LD, but in the observed LD in a given dataset. If an allele is co-occurring with another allele in a data set they will yield similar epistatic interactions regardless of allele frequencies and sample sizes and we therefore use the r^2^ measure of LD [15]. In practice, we calculate LD between variants by sliding linearly along the genome, including variants into blocks as long as the mean r^2^ values between all variant pairs is above a selected threshold. When the blocks are identified, the variant with the highest average r^2^ in the block is selected as a representative for the block.

### Epistatic interaction modelling

The main computational challenge is the calculation of epistatic interactions. Therefore, we have several tools to optimize the calculations of the models. The model used for calculating the epistasis is a heterogeneity model [16, 17]:

$$ y=\mu +{\beta}_1{snp}_i+{\beta}_2{snp}_j+{\beta}_3\left({snp}_i\times {snp}_j\right)+\epsilon $$

Here *y* represents a phenotype of interest, μ is the intercept, *β*_1_ and *β*_2_ are the SNP main effects, *ϵ* is a noise term and most importantly *β*_3_ represents the epsitasis of the two loci. To represent the genotypes *snp*_*j*_ and *snp*_*i*_ we code genotype data as 2 (homozygote minor alleles), 1 (hetrozygote) and 0 (homozygote major alleles). The selection of the values for the genotype affects the model hypothesis. Here there is an assumption of multiplicative interaction between minor alleles in the two sites. We also test for the opposite but mathematically identical model by reversing the minor and major homozygote labels in one of the loci. This test is in case the interaction is between minor and major alleles. There is one more parametrization available in the package, which is 2 (homozygote minor alleles), 1.5 (heterozygote) and 1 (homozygote major alleles). This parametrization tests interaction on the gradient of one allele pair set to the other allele pair, which means that all four alleles are involved in the interaction. This is more powerful description but also more difficult to fit as it requires all four alleles to be related to changes in the phenotype for an optimal fit. In the package there is also a generalized linear model (GLM) implemented so that case-control studies (where case-control are coded in binary form as 1–0) can be analyzed. The GLM version is about twice as slow as the non-binary version, as it fits an underlying liability threshold models. The basic linear model uses implementations linked to underlying C++ code, ensuring fast computations of epistatic interactions. The algorithm is fully parallelized. A test setting is included to test runtimes based on input data and the number of threads used.

### Network and module creation

The original idea of WGCNA was based on using correlations in expression data to find interconnected gene. From there it is a natural extension to genomic interactions in networks, by converting the epistatic estimates (the *β*_3_in the model) to correlations by rescaling them from − 1 to 1. This is done by treating the negative and positive *β*_3_ separately to insure that values close to zero correspond to a correlation of zero. The resulting similarity matrix is then used to calculate the topological overlap measure (TOM) [18]. The next steps follow the workflow of WGCNA: the dissimilarity TOM is used to define modules by creating a gene dendrogram and cutting of branches using a tree-cutting algorithm. Modules are then correlated to the phenotype of interest to detect biologically interesting modules. The functions of WGCNA are integrated in the WISH package for optimization of the workflow. For more details, see Kogleman et al. [13].

### Visualization and result assessment

**Fig. 2:**
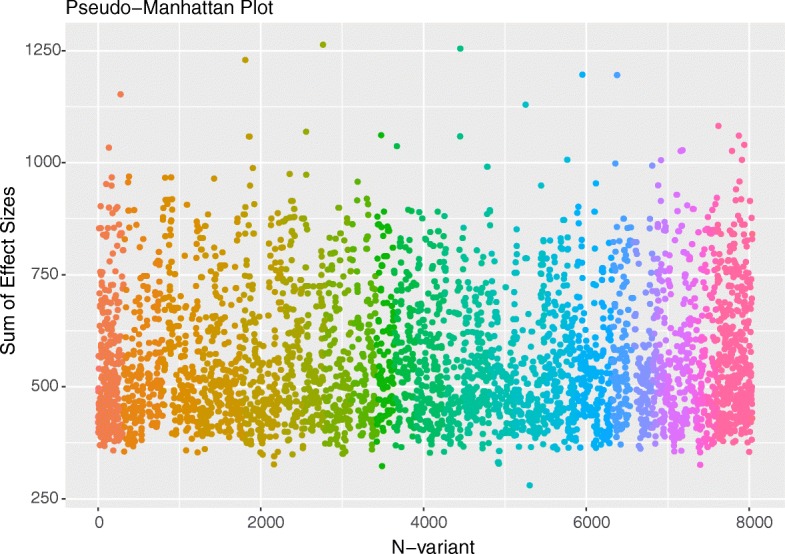
Example of a Pseudo Manhattan plot. Visualizing interactions in a meaningful way is difficult due to the high data dimensionality. One way to solve this is to use summary statistics for each locus instead. Here we sum over the -log likelihoods of all interactions for each variant to give an idea of which variants are most strongly interacting across the genome and color by chromosome

**Fig. 3:**
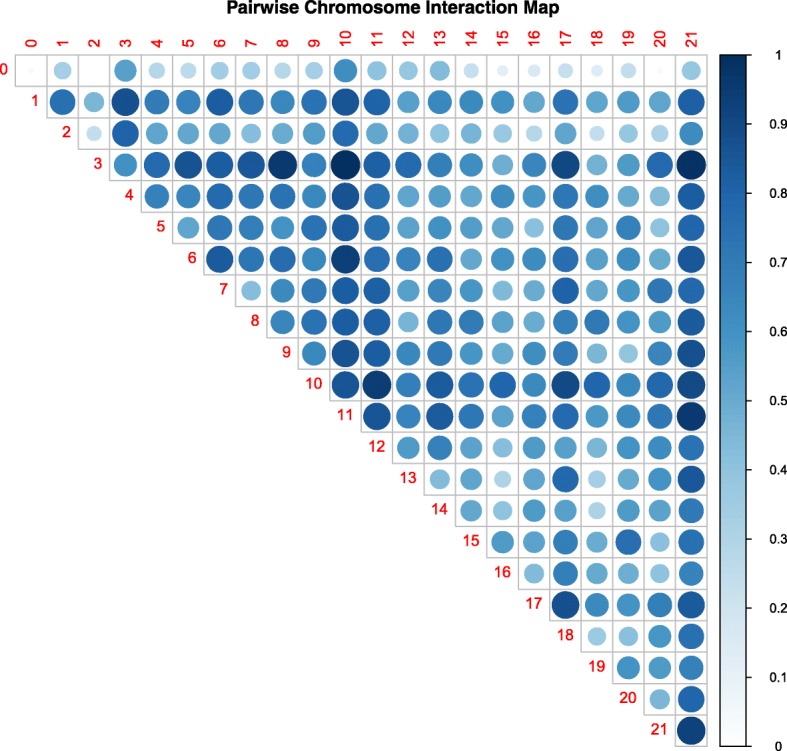
Visualization of pairwise chromosomal interaction strength. Chromosomal interactions are found by calculating the 90th percentile of the –log likelihood of all epistatic interactions between each chromosome pair and then normalizing them to from − 1 (weakest) to 1 (strongest) interactions

Visualizing high dimensional data from epistasis in an informative and meaningful way can be a challenge. In the WISH R package, we have implemented several functions for visualizing and summarizing epistatic interactions. The first method is a pseudo Manhattan plot, based on calculating the sum of -log likelihoods for each variant across all tested interactions. See Fig. 2 for an example. Another measure is a genome wide interaction overview, created by calculating quantile values of significance of interaction between chromosomes, as seen in Fig. 3. While this does not give an accurate representation of individual interactions, it does indicate which chromosomes may be hot spots for interactions for a given phenotype. An example can be seen in Fig. 3. The other option is to visualize epistasis between individual chromosomes. This is done by visualizing the strength of epistasis in all pairwise regions of a user-defined size between selected chromosomes (Additional file 1: Figure S1).

## Results and discussion

### Performance

**Fig. 4:**
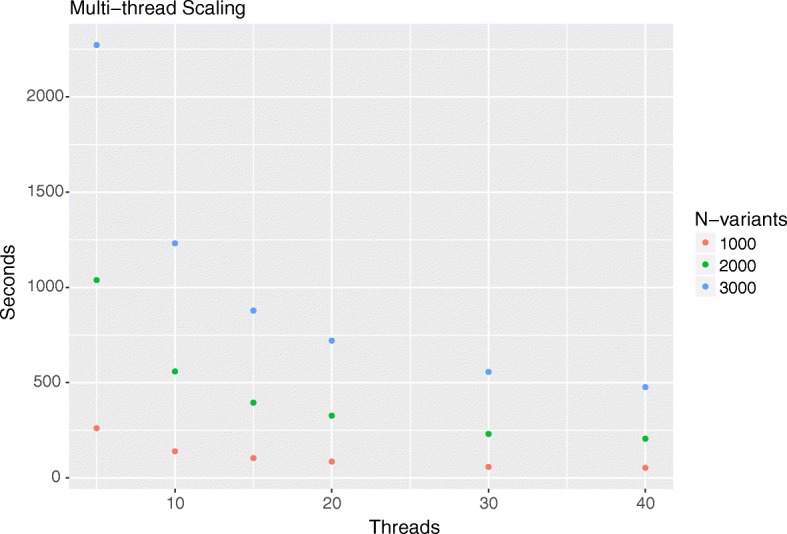
Scaling of runtime using multithreading based on 1000, 2000 or 3000 variants and 500 samples using simulated genotypes and phenotypes. We see that the improvement in run time with increased number of threads is not linear, due to increased overhead. In all the different runs the improvement in runtime from 5 to 40 threads is ab out 5-fold. On the other hand, the number of variants has no effect on the speed with about 9000 models per second being calculated using 40 threads across all runs. This is because as with larger data sets the individual threads handle larger data chunks at a time, leading to less overhead

When dealing with epistasis it is important to have efficient algorithms. We tested the performance of this part of the package using randomly simulated phenotypes and genotypes. In Fig. 4 we can see the runtime of WISH based on different number of variants and 500 samples using different amount of threads. The test where conducted using AMD Opteron 6380 Processors running at 2.5 GHz with varying number of cores used. There is an approximately linear increase in run-speed based on the number of threads. With our benchmark, it would take around 3 h for 10,000 variants or about three days for 50,000 variants using 40 threads. In Additional file 2: Figure S2 we see that the package is not sensitive to the number of samples, and can therefore be run on a wide range of sample sizes. The LD filtering and network analysis part of the package are entirely dependent on the input data, and do not have any computational challenges. For an example of a full analysis see the original WISH paper [13, 19].

### Method comparison

In general, it is difficult to compare methods that calculate epistasis as different models and definitions of epistasis are used. *SNPassoc* [20] can calculate epistatic interactions but lacks any strategies or recommendations for the computational issues. *EPIBLASTER* [21] reports being able to calculate a high amount of interactions but requires a GPU computing facility and specific sub-setting and partitioning of the data. Their strategy to filter the data is to a priori calculate simple correlations between cases and controls and variants, as their method only applies to binary phenotypes. This is similar to our suggested approach of using a main effect filtering, however, they end up calculating much fewer interaction models. They report being able to analyze 300 k markers in one day but, using real data they only calculate actual epistasis in 373,153 SNP pairs out of 4.5 × 10^10^ possible pairs. Their implementation does not include the epistasis modelling, requiring more work to get the epistasis results., *FastEpistasis* [22] has a similar idea as our method for the epistasis calculations, but it only has focus on one aspect, namely calculating the models. They do not discuss filtering strategies or data analysis strategies but are able to calculate the models faster. Martinéz et al. [23] also focus only on epistasis without filtering steps, but report having a higher sensitivity than other available methods, but they do not present any evidence as to why this should be the case. Their implementation has comparable speed to ours. Boost [24] offers very high performance based on using approximated calculations setups, but is not straightforward to use, as it requires non-standard input files and requires specific GPU computing software and hardware setups. Similar to Boost but more recently, Gonzalez-domingues et al. [25] are able to calculate epistasis for large datasets, but they also use specialized hardware setups and it is unclear if their implementation is generally available. We believe that WISH offers several advantages compared to other models. Our method works both on quantitative and binary phenotypes, and we apply the full model to all pairs in the input space. Most of the above methods are able to calculate epistatic interactions at a faster speed than our implementation, but this comes at a cost. Either heuristic filters are applied, or specific hardware is needed, and often the methods themselves are not straightforward to use. In regards to speed, it is unlikely that it is necessary to calculate epistasis for all SNP pairs on a high-density SNP chip, as many of these calculations will be redundant or not biologically related to the trait of interest. The epistasis calculations of WISH should be fast enough to cover most or all biologically relevant SNPs We present strategies for filtering the data using SNP main effect and we include a built-in LD filter, thus ensuring a proper selection of biologically meaningful SNPs. We also implement a solution for dealing with the epistatic coefficients, namely the application of network-based analysis. Epistasis is in general a very complex subject, and the estimation the epistasis itself is just the start of the analysis. Network analysis is the natural extension of pairwise epistasis, as allows us to identify and analyze more complex genomic interaction patterns. One more feature we have that we found lacking in other methods is visualization. Visualizing high dimension epistasis data is technically difficult, but we have included some options for summarized assessment of the epistatic modelling, which we found to be lacking in other methods. Our package is simple to use and implemented in R, making it easy to install, transparent to use, and the outputs are easy to manipulate for the user.

## Conclusions

Epistasis in an important component of genetic variation and may have causal effects in certain diseases or complex trait manifestation in humans, animals, plants and other organisms. However, analysis of epistasis on genome-wide scale is an overlooked subject with several challenges, mainly interpretation and data dimensionality issues. We have previously proposed the WISH method for calculating epistasis and applying the results in a network framework, thus offering solutions for some of the main issues in the analysis of epistasis. Here we have implemented WISH-R, an efficient R package for calculating linear interaction between genomic variants from standard genotype data and generating modules of groups of interacting variants. WISH-R is easy to install and use, and provides tools for analyzing epistasis in complex traits and diseases based on whole genomic data from data filtering to final interpretation.

## Availability and requirements

**Project name:** WISH-R package.

**Project homepage:** https://github.com/QSG-group/wish

**Operating system:** Platform Independent.

**Programming Language:** R.

**Other requirements:** R 3.0 or >.

**License:** GPL-3.

**Restrictions to use by non-academics:** license needed.

## Additional files

Additional File 1: **Figure S1.** Example visualization of the package function *pairwise.chr.map()* function displaying the strength of epistatic interaction between regions on two chromosomes. (DOCX 38 kb)

Additional File 2: **Figure S2.** Visualization of the runtime scaling of the method based on changes in sample size. (DOCX 27 kb)

## Abbreviations

GLM: Generalized linear model
GWAS: Genome Wide Association Studies
IBD: inflammatory bowel disease
LD: Linkage disequilibrium
Ped: pedigree file
TOM: Topological overlap measure
Tped: transposed ped file
WISH: Weighted Interaction SNP hub
WISH-R: The WISH R package
