# Supplementary material for: WISH-R– a fast and efficient tool for construction of epistatic networks for complex traits and diseases
Source: BMC Bioinformatics. 2018 Jul 31;19:277. doi: 10.1186/s12859-018-2291-2 (PMC6069724; doi:10.1186/s12859-018-2291-2)
Supplement: Supplementary file 1 — Figure S1. Example visualization of the package function pairwise.chr.map() function displaying the strength of epistatic interaction between regions on two chromosomes. (DOCX 38 kb) [file 12859_2018_2291_MOESM1_ESM.docx]

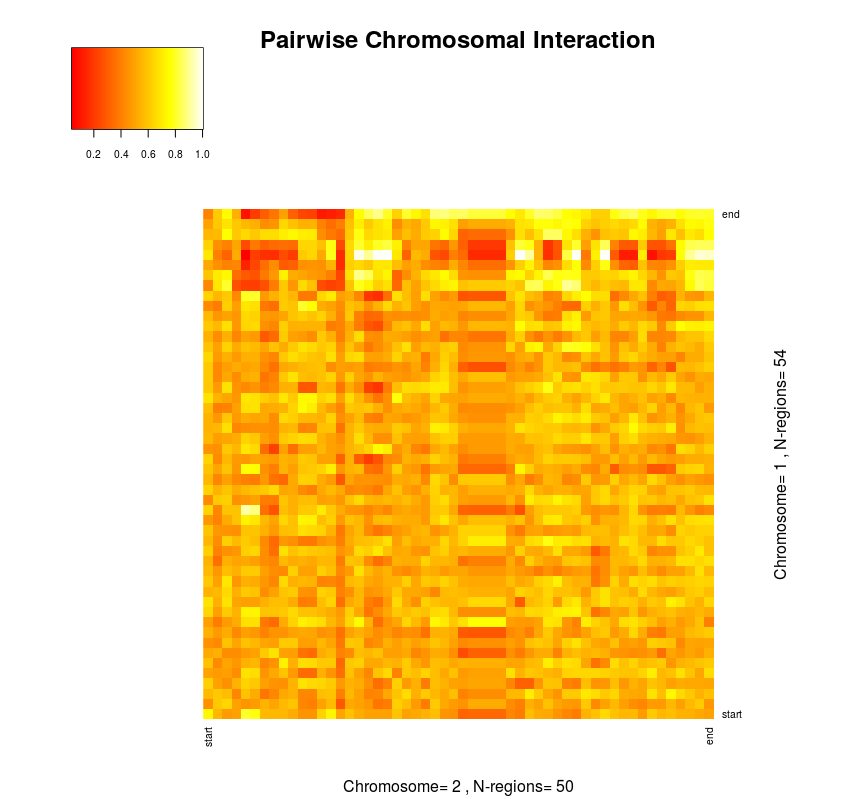


*Figure S1 - Example of Chromosome pairwise region map of strength of epistatic interactions between regions, generated by the pairwise.chr.map() function. Chromosomes are automatically split into suitable regions and linearly compared from start to end indicating which regions are strongly interacting*
