# Supplementary material for: WISH-R– a fast and efficient tool for construction of epistatic networks for complex traits and diseases
Source: BMC Bioinformatics. 2018 Jul 31;19:277. doi: 10.1186/s12859-018-2291-2 (PMC6069724; doi:10.1186/s12859-018-2291-2)
Supplement: Supplementary file 2 — Figure S2. Visualization of the runtime scaling of the method based on changes in sample size. (DOCX 27 kb) [file 12859_2018_2291_MOESM2_ESM.docx]

*
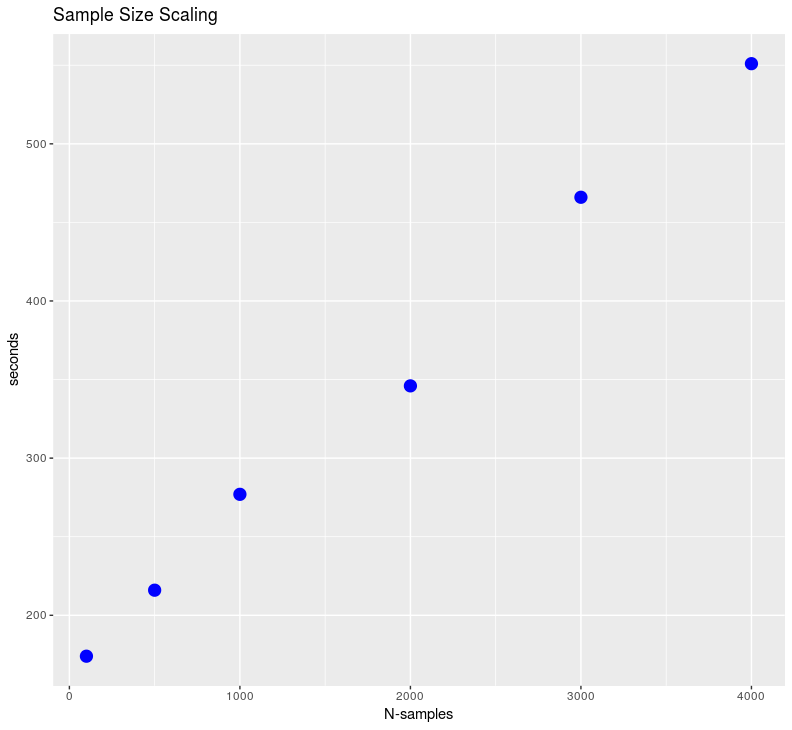
*

*Figure s2 – Scaling of runtime based on change in the number of samples. All runs used 40 threads and 2000 variants. The main conclusion is that the method is not very sensitive to increasing sample sizes, as with a 40-fold increase in the number of samples we only observe roughly a 3-fold increase in runtime.*
